# Reference Gene Selection for Insect Expression Studies Using Quantitative Real-Time PCR: The Head of the Honeybee, *Apis mellifera*, After a Bacterial Challenge

**DOI:** 10.1673/031.008.3301

**Published:** 2008-04-22

**Authors:** Bieke Scharlaken, Dirk C. de Graaf, Karen Goossens, Marleen Brunain, Luc J. Peelman, Frans J. Jacobs

**Affiliations:** ^1^ Laboratory of Zoophysiology, Department of Biochemistry, Physiology and Microbiology, Faculty of Science, Ghent University, Belgium; ^2^ Laboratory of Animal Genetics, Department of Nutrition, Genetics and Ethology, Faculty of Veterinary Medicine, Ghent University, Belgium

**Keywords:** insect immunity, neuro-immunity, housekeeping genes, normalization, variability, transcription

## Abstract

In this study an important and often neglected aspect of gene expression studies in insects, the validation of appropriate reference genes with stable expression levels between sample groups, is addressed. Although in this paper the reference gene selection for the honeybee, *Apis mellifera* L. (Hymenoptera: Apidae) head was tested in the context of bacterial challenge with *Escherichia coli*, this work can serve as a resource to help select and screen insect reference genes for gene expression studies in any tissue and under any experimental manipulation. Since it is recommended to use multiple reference genes for accurate normalization, we analyzed the expression of eleven candidate reference genes in the honeybee head, for their potential use in the analysis of differential gene expression following bacterial challenge. Three software programs, BestKeeper, Normfinder and geNorm, were used to assess candidate reference genes. GeNorm recommended the use of four reference genes. Both geNorm and Normfinder identified the genes *GAPDH, RPS18, actin* and *RPL13a* as the most stable ones, only differing in their ranking order. BestKeeper identified *RPS18* as being the reference gene with the least overall variation, but also *actin* and *GAPDH* were found to be the second and third most stable expressed gene. By a combination of three software programs the genes *actin, RPS18 and GAPDH* were found suitable reference genes in the honeybee head in the context of bacterial infection.

## Introduction

At present, the most sensitive and accurate method to determine small deviations in mRNA expression levels of a single gene is quantitative real-time PCR. Normalization of real-time PCR data is critical for a reliable mRNA quantification. The most common way to perform normalization is to relate the mRNA level of the gene of interest to the mRNA of a reference gene whose expression level is considered stable, regardless of cell type and across various experimental conditions (Thellin et al. 1999).

For Hymenoptera, and more particularly for the honeybee, *Apis mellifera* L. (Hymenoptera: Apidae) most gene expression studies using quantitative real-time PCR incorporate only one reference gene as an internal control with the reference gene of choice being *actin* (Zufelato et al. 2004; Chen et al. 2005; Lourenco et al. 2005), *RPS5* (Wheeler et al. 2006), *eukaryotic initiation factor S8* (*EIF-S8*; Hunt et al. 2007) and *28S rRNA* (Judice et al. 2006). However, this is not restricted to bee research alone. Suzuki et al. (2000) described that in 1999 over 90% of the RNA transcription analyses published in high impact journals used only one reference gene. Vandesompele et al. (2002) demonstrated that errors in expression data up to 20-fold can be generated by the use of only a single reference gene. According to Thellin et al. (1999) and Vandesompele et al. (2002), at least two or three reference genes should be used for accurate normalization. Moreover, as several studies have shown that reference genes used for the quantification of mRNA expression can vary with experimental set-up and/or cell type (Thellin et al. 1999; Sturzenbaum and Kille 2001), each candidate reference gene should be validated before use to make sure it is stably expressed in a particularly tissue under the given experimental manipulation.

Statistical algorithms such as geNorm (Vandesompele et al. 2002), Normfinder (Andersen et al. 2004) and BestKeeper (Pfaffl et al. 2004) have been developed to assess the appropriateness of reference genes. However, there are no scientific reports comparing the use of these three freely available Excel-based tools for evaluation of the stability of reference genes in the context of expression studies in Hymenoptera. GeNorm, a Visual Basic Application for Microsoft Excel, determines the expression stability of candidate reference genes by assigning each gene a gene-stability measure M. This measure is based on the principle that the expression ratio of two ‘ideal’ reference genes should be identical in all samples, regardless of experimental set-up or cell-type. A value for M is assigned by pair-wise variation for each combination of candidate reference genes. The gene with the highest M-value (i.e. least stable) gets eliminated until the two most stable expressed genes remain. Candidate reference genes are ranked according to the average M-value, an optimal number of reference genes is determined and a normalization factor can be derived based on geometric averaging of the expression level (relative quantities) of the most stable reference genes. Normfinder is another Visual Basic Application, which also assigns a stability value to each candidate reference gene. This value ranks the genes using a model based-approach (mixed linear effect modeling). Instead of analyzing the expression of the whole data set, as is the case with geNorm, this program focuses on the inter-and intra-group expression variations. The Excel-based tool BestKeeper analyzes variability in expression of candidate reference genes by calculation of cycle threshold (Ct) data variation (standard deviation and coefficient of variation). Next the software performs comparative analysis based on numerous pair-wise correlations of all candidate reference genes against each other. The relation between BestKeeper index and the contributing reference genes is further defined by pair-wise correlation analysis.

The aim of this work is to address an important and often neglected aspect of gene expression studies in the honeybee as well as in other insects: the validation of appropriate reference genes with stable expression levels between sample groups. Although in this paper the reference gene selection for the honeybee head was tested in the context of bacterial challenge with *Escherichia coli* (Scharlaken et al. 2007), this work can serve as a resource to help select and screen insect reference genes for gene expression studies in any tissue and under any experimental manipulation.

## Materials and methods

### Sample collection

Newly emerged (up to 1 day old) Carniolan honeybee workers, Apis *mellifera carnica*, were collected from hives of the experimental apiary in Ghent. Two groups of 50 worker bees were created. The first group was pricked in the abdomen (between 2 and 3 tergite) with a sterile needle dipped in a bacterial suspension of *E. coli* NCTC 9001 (three fresh colonies, overnight grown on nutrient-agar plate, suspended in 500 µl sterile physiological solution containing 15 mM NaCl, 75 mM KCl, 3 mM CaCl_2_, 10 mM MgCl_2_, 55.5 mM glucose, 15 mM sucrose and 55.5 mM fructose). The second group was pricked in the same way with a sterile needle dipped in the described physiological solution. After pricking, the two groups were put in laboratory cages and incubated at 34°C and 70% RH, with ad libitum water and sugar water.

**Table 1.**
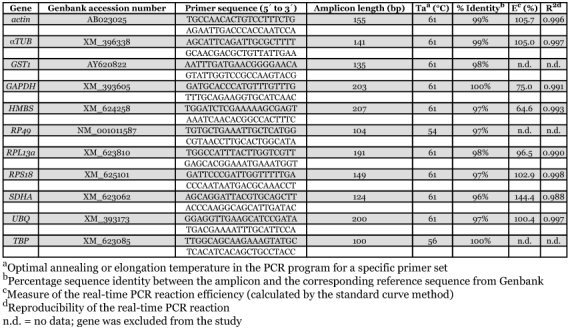
Information of the primers used for real-time PCR.

Eight hours after bacterial challenge, animals were anesthetized by chilling. In both groups the whole head of each worker was separated from the body by cutting precisely at the end of the thoracal tagmatum using a pair of scissors. Each whole head was stored separately in 250 µl RNA*later* solution (Ambion,www.ambion.com) at -20°C.

### RNA extraction and cDNA synthesis

Total RNA was isolated from each whole head using the RNeasy Mini kit (Qiagen, www.qiagen.com) according to the manufacturer's protocol. For genomic DNA removal, an on-column DNase digestion with the RNase-free DNase set (Qiagen) was carried out according to the manufacturer's instructions. RNA was eluted using 40 µl RNase-free water and stored at -80°C. A minus RT control with primers for *actin* (Cunha et al. 2005) was performed to check for successful removal of all contaminating DNA.

First-strand cDNA was synthesized from 5 µl total RNA (10 ng-5 µg) using the RevertAid H Minus First Strand cDNA synthesis kit (Fermentas, www.fermentas.com) following the manufacturer's instructions. The kit's reverse transcriptase is a genetically engineered version of the Moloney Murine Leukemia Virus reverse transcriptase, and is used for first strand cDNA synthesis at the 3′-end of poly(A)+ mRNA's using the oligo(dT)18 primer. The cDNA of the honeybee head was diluted 5 times with Tris-HCl (pH 8, 10 mM).

### Reference gene selection and primer design

Eleven reference genes were selected (see Table 1). Primers for *actin* were used from Cunha et al. (2005). Based on already described mammalian and insect reference genes in literature, the NCBI (http://www.ncbi.nlm.nih.gov) and Glean3 databases (Honeybee Genome Sequencing Consortium 2006) were used to search for available honeybee sequences for the other ten reference genes: α*TUB* (Vontas et al. 2000), *RPS18* (Donnell et al. 2006), *GST1* (Justice et al. 2003), *RP49* (Iovchev et al. 2006), *UBQ* (Mittapalli et al. 2006), *RPL13a, GAPDH, HMBS, SDHA* and *TBP* (Vandesompele et al. 2002). Except for *RP49* and *GST1*, the honeybee sequences were based on *in silico* predictions and not yet experimentally confirmed (see Table 2). We designed primers in sequences coding for conserved protein domains using Primers software (http://frodo.wi.mit.edu/cgi-bin/primer3/primer3_www.cgi), taking into account the possible secondary structures of the amplicon (Mfold software) (http://www.bioinfo.rpi.edu/applications/mfold/cgi-bin/dna-form1.cgi). Primer conditions were optimized by determining the optimal annealing temperature (Ta) and primer concentration (3.125 µM). Primer and amplicon information are listed in Table 1. Amplicons were sequenced for verification using a Perkin Elmer (www.perkinelmer.com) Applied Biosystems 3130XL automated DNA sequencer with 50 cm capillaries filled with POP-7 polymer. PCR product was treated with shrimp alkaline phosphatase (1 U/µl, Amersham Biosciences, www.amersham.com) and exonuclease I (20 U/µl, Epicentre Technologies, www.epicentre.com/main.asp) for 15 minutes at 37°C, followed by 15 minutes at 80°C to inactivate the enzymes. This material was then used for cycle sequencing without any further purification, using the ABI Prism BigDye V 3.1 Terminator Cycle Sequencing kit.

**Table 2.**
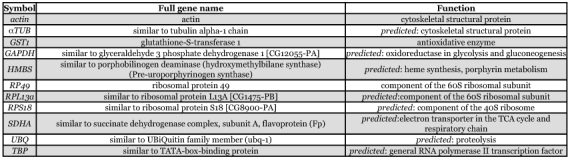
Function of the selected reference genes.

### Real-time quantitative PCR

All real-time quantitative PCR reactions were performed on the iCycler iQ Real-Time PCR detection system (Bio-Rad, www.bio-rad.com). Each 15 µl reaction consisted of 7.5 µl Platinum SYBR Green qPCR SuperMix UDG (uracil-N-glycosylase; Invitrogen, www.invitrogen.com) spiked with 0.15 pmole fluorescein calibration dye (Bio-Rad), 2.5 µl diluted cDNA, the optimized amount of forward and reverse primer and 3.8 µl water (Molecular Biology Grade, Eppendorf, www.eppendorf.com). The PCR program consisted of an initial 2 minute UDG incubation step at 50°C, followed by a 2 minute denaturation at 95°C. Next, 45 cycles consisting of 20 seconds of denaturation at 95°C and 40 seconds of annealing at the optimal annealing temperature during which fluorescence was measured. This was followed by the measurement of fluorescence during a melting curve in which the temperature raised from 70 to 95°C in sequential steps of 0.5°C for 10 seconds. This insured the detection of one gene-specific peak and the absence of primer dimer peaks. Three negative controls and a 5-fold dilution series of pooled cDNA were included in each run. This pooled sample consisted of cDNA from heads from both groups (bacteria-challenged / control). The 5-fold dilution series were used to construct a relative standard curve to determine the PCR efficiency. PCR efficiencies were used to convert cycle threshold values (Ct-values; the fractional PCR cycle at which the fluorescent signal significantly rises above the background signal) into raw data (relative quantities). Each reaction was run in duplicate, whereby three negative controls were included.

### Determination of reference gene expression stability

To determine the expression stability of the selected reference genes in a honeybee head, mRNA expression of the reference genes was measured in 10 separate randomly selected heads of the bacteria-challenged group (biological replicates) and in 10 separate randomly selected heads of the control group (biological replicates). Each individual head reaction was run in duplicate (technical replicates). This means that for each reference gene, 48 real-time PCR reactions were performed, including three negative controls and a 5-fold dilution series with 5 measuring points. Next, in order to compare the transcription level of the selected genes across different samples and experimental manipulation, the average Ct-value of each duplicate reaction was converted to raw data for subsequent analysis with the geNorm (Vandesompele et al. 2002) and Normfinder (Andersen et al. 2004) programs. The average Ct-value of each duplicate reaction was also used (without conversion) to analyze the candidate reference genes with BestKeeper (Pfaffl et al. 2004)

**Figure 1.**
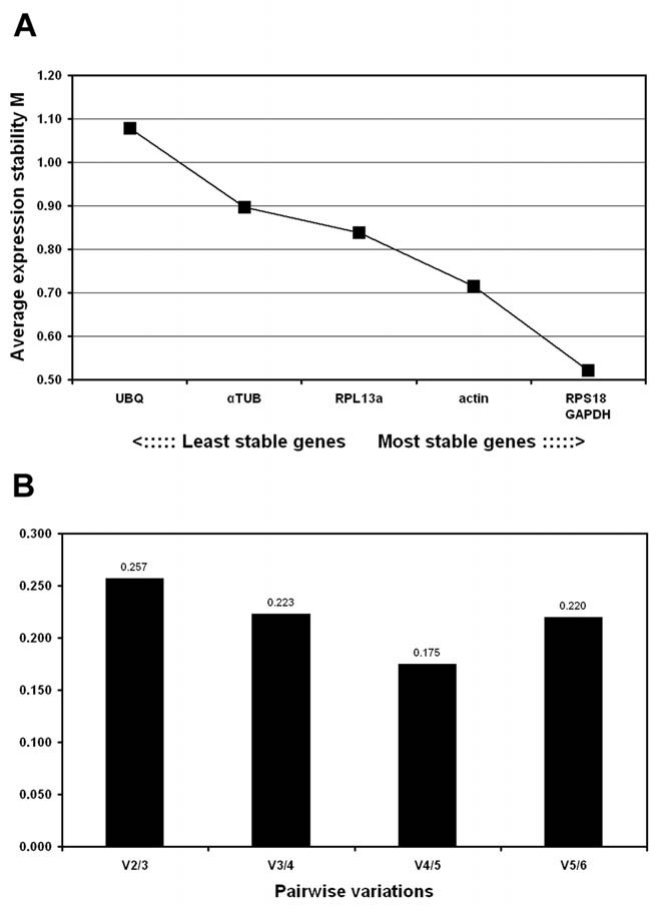
Gene expression stability of the candidate reference genes analyzed by the geNorm software based on the principle that gene pairs that have stable expression patterns relative to each other are appropriate reference genes. (A) Average expression stability values (M) of the six remaining reference genes plotted from least stable (left) to most stable (right). (B) Pair-wise variation analysis between the normalization factors NF_n_ and NF_n+1_, to determine the optimal number of reference genes for normalization. The value V5/6 is higher than V4/5; this is due to the inclusion of a relative unstable fifth gene. Increasing variation in this ratio corresponds to decreasing expression stability.

## Results

### Transcription profiling of candidate genes

Initial screening of eleven potential reference genes by reverse transcriptase-PCR showed that all the genes were expressed in the honeybee head, visualized by a single amplicon with the expected size on a 2% agarose gel. All these amplicons were sequenced for verification and all displayed identity >96% with the described sequences on which primer design was based (Table 1). Due to the formation of primer-dimers (for *RP49, GST1* and *TBP*) and a weak single amplicon (*RP49* and *TBP*), these three genes were excluded from the study. Primer dimer formation and unspecific amplification can falsely increase gene expression levels and must be avoided, especially when performing real-time PCR using SYBR green intercalating dyes. Gene-specific amplification of the other eight genes in the head was confirmed by a single peak in real-time melt-curve analysis. A standard curve was generated for each gene in the head, using the 5-fold serial dilutions of pooled cDNA, generated form infected and non-infected bee heads. The correlation coefficient (R^2^) and PCR efficiency (E) characterizing each standard curve are given in Table 1. PCR efficiencies of the amplification of the eight genes in the honeybee head displayed for most genes very good PCR efficiencies (varying from 64.6% to 144.4% of which five varied between 96.5% and 105.7%). Due to unacceptable PCR efficiencies the genes *HMBS* (64.6%) and *SDHA* (144.4%) were also excluded from the study.

### GeNorm analysis

The ranking of the six candidate reference genes in whole head according to their average expression stability (M value) is shown in Figure 1A. From the most stable (lowest M-value) to the least stable (highest M value): *GAPDH/RPS18* < *actin* < *RPL13a* < *αTUB* < *UBQ*. All six genes reach high expression stability with low M-values, below the default limit of M = 1.5 (Table 3). GeNorm analysis revealed that the pair-wise variation value V5/6 is higher than V4/5 (Figure 1B). Increasing variation in this ratio corresponds to decreasing expression stability, due to the inclusion of a relatively unstable fifth gene. So four genes (*GAPDH, RPS18, actin* and *RPL13a*) are necessary for accurate normalization. Including a fifth reference gene has no significant effect on the normalization factor.

**Table 3.**
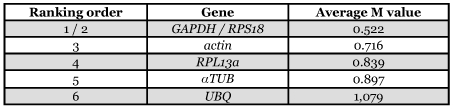
Candidate reference genes for normalization ranked according to their expression stability (calculated as the average M Value after stepwise exclusion of the worst scoring gene) by geNorm.

### Normfinder analysis

The four most stable expressed reference genes are identical to the ones previously determined using geNorm but the ranking order is different (Table 4). From most stable (lowest stability value) to least stable (highest stability value): *actin* < *RPL13a* < *GAPDH* < *RPS18* < *αTUB* < *UBQ*. The ranking of the two least stable expressed genes is identical between geNorm and Normfinder.

**Table 4.**
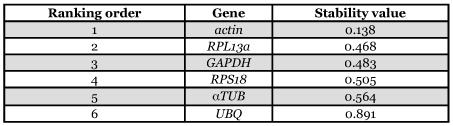
Candidate reference genes for normalization listed according to their expression stability calculated by NormFinder.

### BestKeeper analysis

Gene expression variation was calculated for all six candidate reference genes based on Ct-values and displayed as the standard deviation (S.D.) and coefficient of variance (C.V.). BestKeeper highlighted *RPS18* as the reference gene with the least overall variation from the list of six candidate genes (Table 5), with an S.D. of 0.72, which represents an acceptable 1.71-fold change in expression. The variation in expression of the other candidate reference genes was greater than two-fold (S.D. greater than 1.0). From most stable (lowest S.D.) to least stable (highest S.D.): *RPS18* < *actin* < *GAPDH* < *αTUB* < *UBC* < *RPL13a*. Next, the pair-wise correlation between genes and the correlation between each gene and the BestKeeper index were calculated. The best correlation between BestKeeper index and candidate reference gene (Table 6) was obtained for *actin* (r = 0.976) and *RPL13a* (r = 0.967), which were also ranked as the two most stable expressed reference genes using Normfinder.

## Discussion

Normalization of gene expression is used to correct sample-to-sample variation in order to identify real gene-specific variation. Normalization of mRNA levels to cell number is not possible when using whole tissue samples like the head (Vandesompele et al. 2002). Therefore, real-time PCR data are usually normalized against a control gene. Traditionally, genes thought to have stable expression in the honeybee such as *actin* and *28S rRNA*, have been employed. Due to increased sensitivity and dynamic range of real-time PCR over traditional quantification techniques, the expression of several so called housekeeping genes has been shown to vary with treatment, biological process and/or tissue or cell type.

Given the described disadvantages, a new method of employing multiple reference genes has emerged which determines the expression stability of different reference genes in samples of interest. Three freely available software programs (geNorm, Normfinder and BestKeeper) were used to evaluate the expression stability of candidate reference genes. The present paper demonstrates that these tools permit setting up a validation procedure with a broad range of candidate reference genes consisting of known honeybee reference genes (such as *actin*), next to the homologues of reference genes from closely (wasp: *RPS18*) and distantly related (human: *GAPDH* and *RPL13a*) animal species. Although only *actin* was incorporated in this study as known honeybee reference gene, it is obvious that other known reference genes can be included and re-evaluated in further studies. With the genome sequence of the honeybee available, it seems possible to implement in such screening protocols, genes of the honeybee from which functional information is still lacking. In this way the array of candidate honeybee reference genes for further experiments is broadened significantly.

**Table 5.**
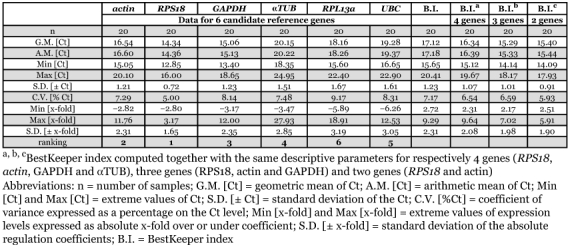
BestKeeper descriptive statistical analysis for reference genes based on cycle threshold (Ct) values

**Table 6.**
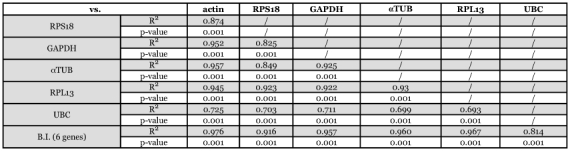
Repeated pair-wise correlation analysis of candidate reference genes. Genes are pair-wise correlated one with another and then with the BestKeeper index.

The elimination of the genes *GST1, TBP, RP49, SDHA* and *HMBS* from this analysis due to primer dimer or efficiency problems does not implicate that these genes are unsuitable as reference genes. Primer dimer formation is due to sequence complementarity of the forward and reverse primers of a given gene and can be solved by designing a new primer set. Low efficiency can be related to the tissue examined or the experimental set-up, but does not necessarily mean that the same primer set is unsuitable to normalize data sets from another physiological study.

This study only tested the suitability and problems in reference gene selection in conjunction with the DNA binding dye SYBR Green. This fluorescent dye binds in the minor groove of double-stranded DNA in a sequence-independent way. Another fluorescent method allows specific sequence detection because of the use of fluorescently sequence-specific hybridization probes. Several types of probes (TaqMan, Molecular Beacons, LightCycler and Amplifluor) can be used. Some of the SYBR Green-related problems (formation of primer dimers; formation of secondary structures in the PCR product; primer concentration, which can be limiting) are not as important when using these sequence-specific probes. However, unwanted products can be formed but remain undetected, which may alter the amplification efficiency of the specific product. When using SYBR Green, non-specific products can be detected as separate distinct melting peaks in a melting curve analysis. Both detection formats (SYBR Green and hybridization probes) are potentially rapid and sensitive. Therefore we preferred the use of the more economical SYBR Green.

**Figure 2.**
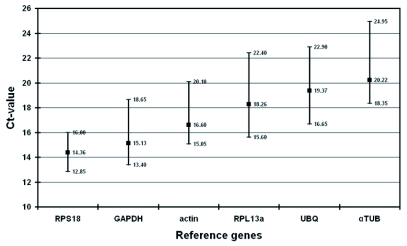
Expression levels of candidate reference genes. Values are given as cycle threshold (Ct) numbers. Boxes represent mean Ct-values and whiskers the range of Ct-values in 20 samples.

The six candidate reference genes used for the data analysis display a relatively wide range of expression level (see Figure 2), from the lowest mean Ct-value (14.36) in *RPS18* to the highest (20.22) in *αTUB*. These six genes were used to compare three different programs (geNorm, Normfinder and BestKeeper). The results from geNorm and Normfinder can be easily compared because they both use raw data (relative quantities) as input data. Both programs identified the same four reference genes (see Table 3 and 4) as most stable expressed, only differing in their ranking order. However, only geNorm provides information about the optimal number of genes in a given experiment, whereas Normfinder gives additional information about the inter- and intra-group expression variations. BestKeeper, on the other hand, uses Ct-values as input data. This perhaps explains the slightly different output when compared to geNorm and Normfinder. According to BestKeeper only the gene *RPS18* can be considered as displaying consistent expression (S.D. < ± 1 Ct). The computed BestKeeper index (B.I.) for the three most stable genes, *RPS18, actin* and *GAPDH*, is acceptable with S.D. = 1.01 (Table 5), implicating that they can be used for normalization. A comparative evaluation of the six candidate reference genes by pair-wise correlation revealed strong correlations (0.916 < r < 0.976) between five of the six candidate genes (Table 6). BestKeeper ranked *RPL13a* as least stable expressed gene, but correlations between the BestKeeper index and candidate reference genes (Table 6) displayed the second best value for *RPL13a*. (r = 0.967). From the three programs, *actin, RPS18* and *GAPDH* can be considered as best reference genes. First, because all three were found to be most stable reference gene by each program. In addition, these three reference genes show the highest expression in the honeybee head (mean Ct-value below 19) of the six candidate genes (see Figure 2).

RTPrimerDB primer & probe database is a public database for primer and probe sequences used in real-time PCR assays created by Vandesompele and colleagues (Pattyn et al. 2003; http://medgen.ugent.be/rtprimerdb/). Submitting primers for evaluated reference genes can prevent time-consuming primer design and experimental optimization, and introduces a certain level of uniformity and standardization among different laboratories. The primers for *GAPDH, RPS18, actin* and *RPL13a*, the four best suitable reference genes according to geNorm (Vandesompele et al. 2002), have been submitted to the RTPrimerDB. Five other primer sets from insect origin (*Drosophila melanogaster*) were already present in RTPrimerDB.

In conclusion, this is the first detailed study on the evaluation of selected reference genes in the honeybee. Caution is advised when using a single reference gene, which is believed to have stable expression in a particular experimental set-up. By a combination of three software programs for data analysis, this study showed that the genes *actin, RPS18 and GAPDH* are the most stable expressed reference genes in the honeybee head in the context of bacterial infection with *E. coli*. It is also important that the reference genes selected should only be used in the same biological context and tissue. Other uses should be re-evaluated.

## Abbreviations

Ct -: cycle threshold
RT -: reverse transcription
